# Intestinal duplications: incidentally ileum duplication cyst in young female

**DOI:** 10.1259/bjrcr.20180077

**Published:** 2019-04-03

**Authors:** Chiara Martini, Paolo Pagano, Gennaro Perrone, Paolo Bresciani, Paolo Dell'Abate

**Affiliations:** 1 Diagnostic Department, University Hospital of Parma, via Gramsci 14, 43100 Parma, Italy; Emergency Surgery, University Hospital of Parma, via Gramsci 14, 43100 Parma, Italy

## Abstract

Gastrointestinal tract duplication is a rare congenital malformation in young patients and in adults, that occur anywhere from the mouth to the anus and their macroscopic structure may be cystic or tubular.

Intestinal duplication does not show specific symptoms, indeed they can present with a variety of symptoms including abdominal distension and pain, sickness, hemorrhage, chronic respiratory disorders, as well as non-painful abdominal mass. Nonetheless, intestinal duplication can remain completely asymptomatic and be diagnosed as an incidental finding. Presentation with acute complications such as intestinal invagination or mechanical occlusion is quite rare.

We present a case of asymptomatic ileum duplication cyst in young female who referred to the emergency department for trauma and was screened by eco-Focus Assessment Sonography for Trauma (eco-FAST), followed by MR and CT.

The patient underwent ileal resection and prophylactic appendicectomy with ileo-cecal termino-lateral anastomosis. In this case, the intestinal duplication cyst was an asymptomatic incidental finding.

## Case presentation

A 28-year-old female referred to the emergency room following a cycle fall caused by an automobile crash.

Physical examination and laboratory work-up were normal. The patient complained slight generalized abdominal pain after the trauma. Pathological and physiological anamnesis were unrevealing.

## Investigation

**Figure 1.**
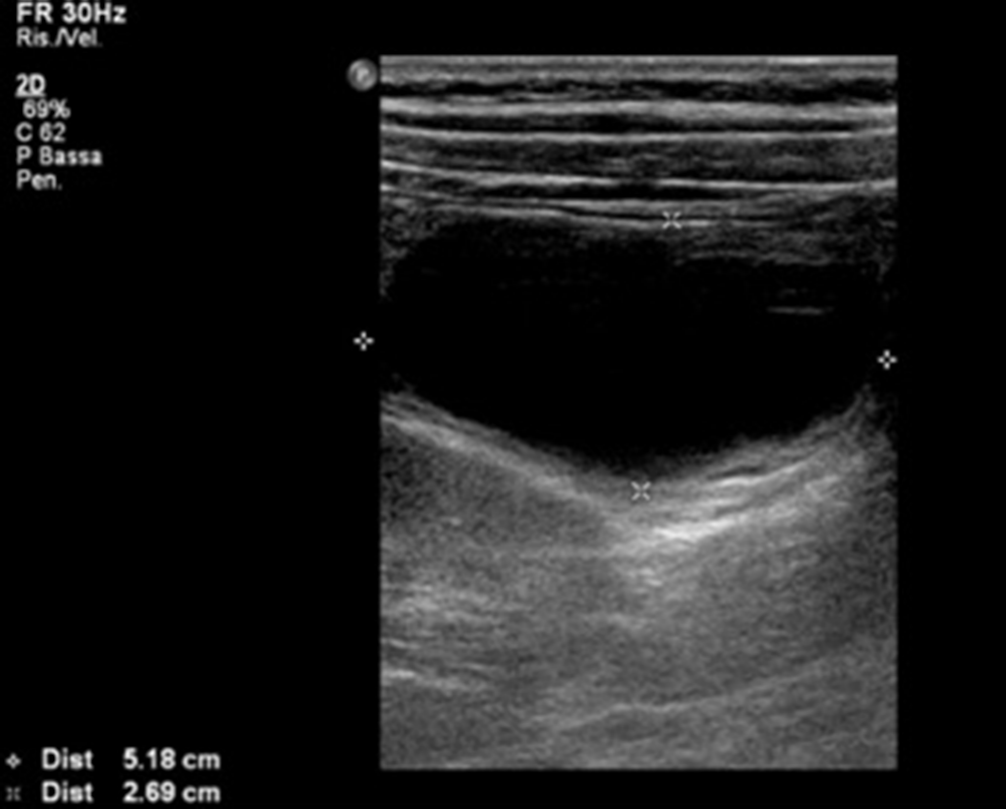
The eco-FAST shows formation of 51 x 27 mm with mainly anechoic content, equipped with a triple-layer wall, movable on the deep planes. FAST, Focus Assessment Sonography for Trauma.

An eco-FAST was performed without signs of traumatic wound, yet with evidence of a right-sided round formation (51 × 27 × 45 mm) with mainly anechoic content, characterized by a triple-layer wall, adherent to an ileal loop, movable on the deep planes and without signs of vascularization (Figure 1). The finding was interpreted as possible expression of intestinal duplication, thus MR was suggested for differential diagnosis.

**Figure 2.**
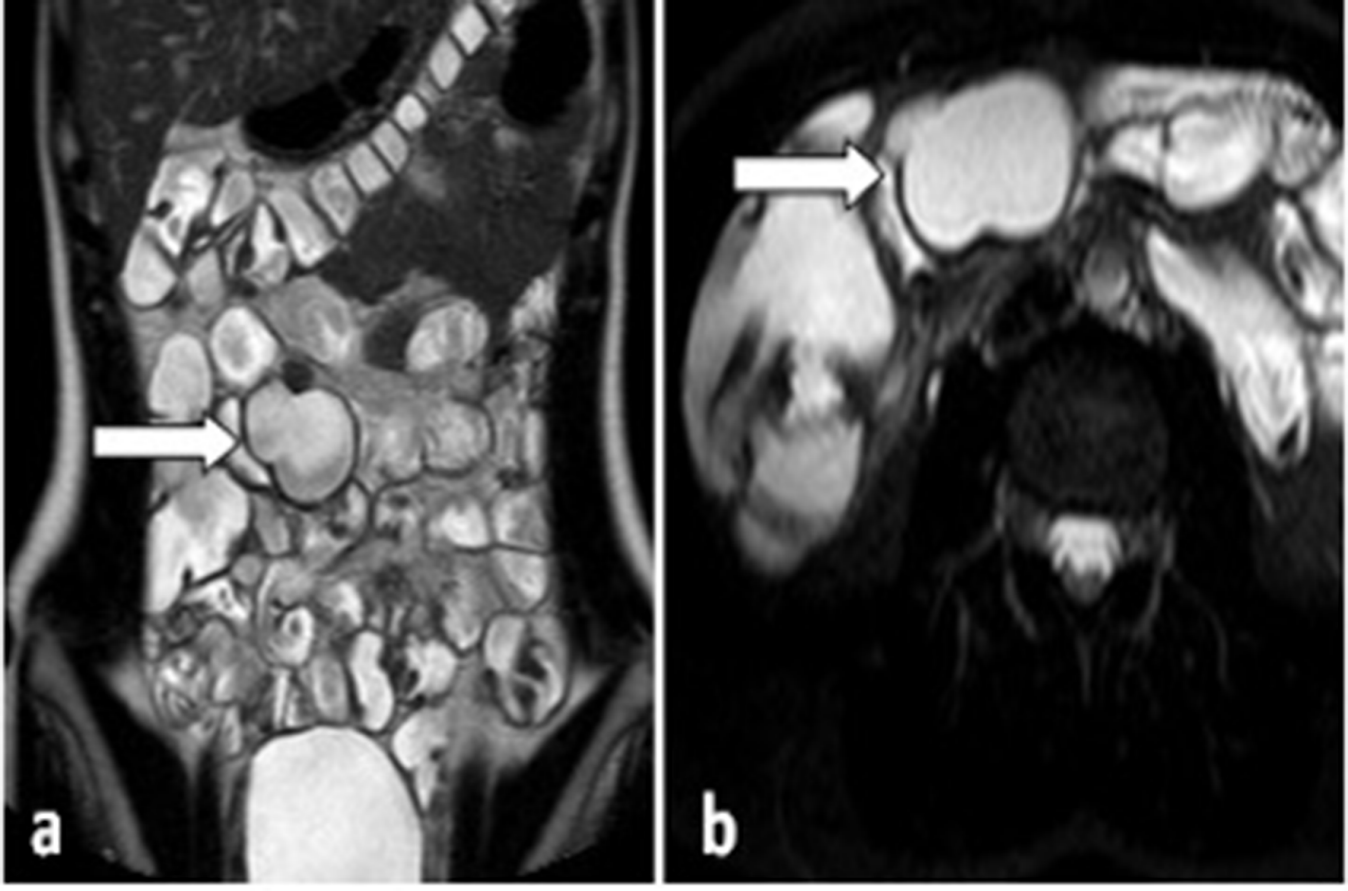
Coronal (a) and Axial (b) MR *T*_2_W images show in the right paramedian intra-peritoneal abdominal site, a polylobate formation of about 52 × 35 × 26 mm characterized by regular margins and hyperintense homogenous content in *T*_*2*_W (white arrow), probably cystic without burials or endoluminal projections.

MR sequences performed were: *T*_2_ weighted (*T*_2_W) SS-turbo spin echo (TSE) BH in coronal and axial plane with and without fat suppression; SSFP-BH in coronal, sagittal and axial plane; *T*_1_W GE three-dimensional with fat pre- and post-contrast media (arterial, venous and tardive phase). The MR confirmed and further characterized the ultrasound finding in the right paramedian intraperitoneal abdominal site. Notably, a polylobate formation (52 × 35 × 26 mm) was observed with regular margins, its walls were characterized by smooth signal intensity and contrast enhancement (comparable to the neighboring intestinal loops) and hyperintense homogeneous content in *T*_2_W and hypointense in *T*_1_W. The lesion showed a posterior peduncle inseparable from distal intestinal loop (probably the last ileal loop, about 10 cm from the ileocecal valve). The finding was deemed compatible with mesenteric duplication cyst (Figure 2).

The patient underwent surgical consultation that placed indication for elective surgery.

## Treatment

Before surgery, the patient underwent also CT and further pre-operative laboratory work-up, which confirmed the surgical option.

The intervention was performed in laparoscopy: the known neoformation was identified in correspondence with the last ileal loop near the ileocecal valve. Ileal resection with end-lateral ileocecal anastomosis and prophylactic appendectomy were performed. The post-operative course was regular and the patient was discharged 7 days after surgery.

**Figure 3.**
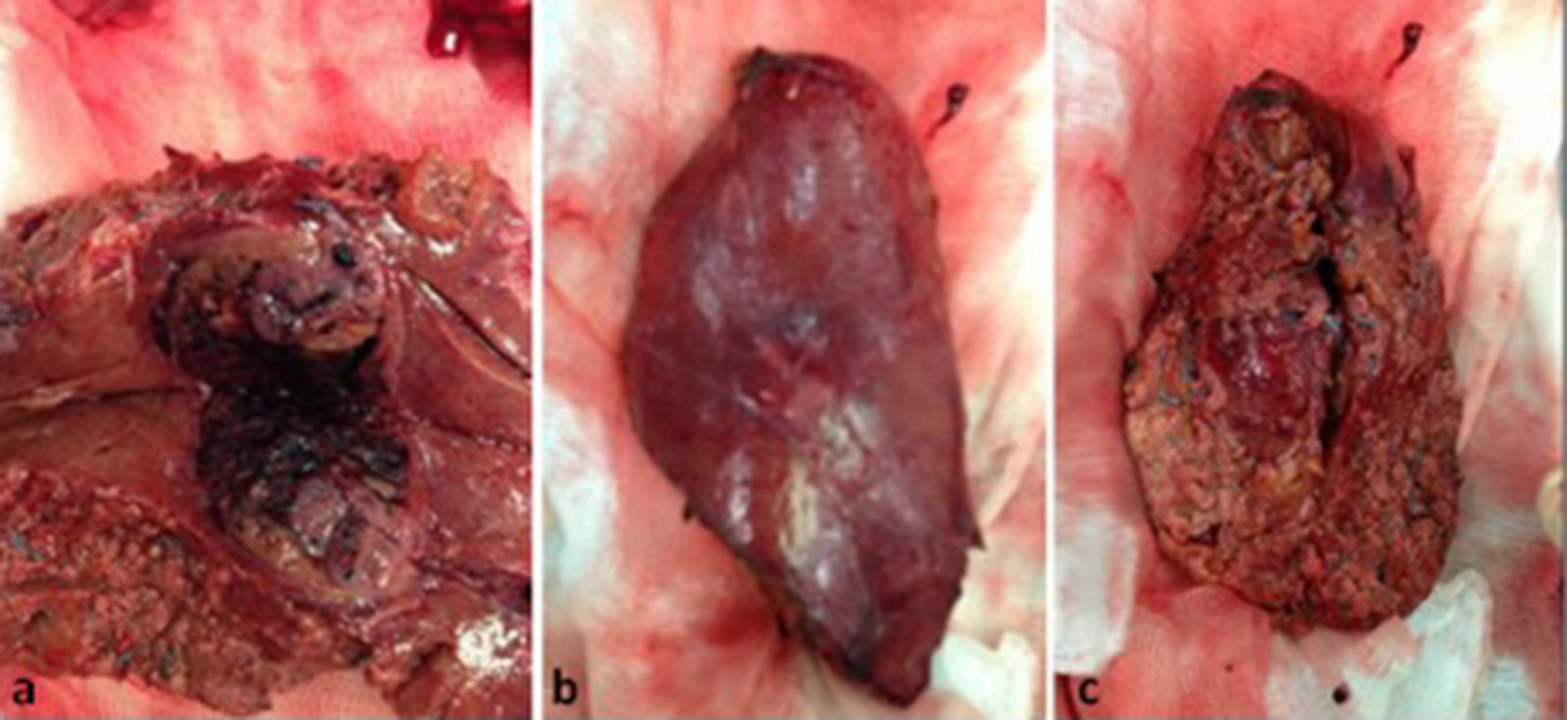
Histological examination (a) and the neoformation is identified in correspondence with the last ileal loop near the ileocecal valve (b) ileal duplication cyst of about 50 x 35 mm of diameter with regular and luminal wall, respectively (c).

The histological examination documented ileal duplication cyst (5 × 3.5 cm) with a smooth wall made up of regular ileal and muscular mucosa, the duplication was not communicating with the intestinal lumen (Figure 3).

The subsequent 1-year control performed with MR did not reveal clinically relevant findings.

## Discussion

The alimentary canal duplications are a group of congenital malformations throughout the whole gastrointestinal tract, from the mouth to the anus.^1^ The term duplication was introduced by Ladd in 1937 in an attempt to group under a single diction all the definitions previously used as “enterogenic cysts”, “enteric cysts”, “double ileum”, “giant diverticula”, “anomalous Meckel diverticula". The same nomenclature was later proposed by Grass in 1952, and it is still referred to. In 1961 Mellish and Koop defined enteric duplications “as spherical or tubular structures that possess a characteristic mucosa of the alimentary canal supported by muscular and serous layers”.^2^

They are very rare and include 0.1–0.3% of all congenital malformations^3^; their incidence is 1:4,500 born, they are mostly located at the jejunoileal level (45%); there are no reported differences in gender distribution (Table 1).

**Table 1.** Locations of duplication locations of the digestive tract and their frequency

Esophageal	19%
Thoracoabdominal	4%
Gastric	9%
Duodenal	4%
Jejunum	10%
Ileal	35%
Cecum	5%
Colon	7%
Rectum	5%

Several theories have been proposed to explain the origin of these lesions, however none of these adequately explains all known duplications. Bentley and Smith in 1960 proposed the notorious disruption theory to describe the many abnormalities involving the spine, the gastrointestinal tract and the skin at the mediastinal level. This theory support the presence of endodermal defect that results from altered separation of the notochord during the presomitic stage of embryonic development. Other authors proposed a non-regression of those embryonic diverticula that are regularly present during intrauterine development (*e.g,* diverticula of the stomach, of the duodenum and of the ileum). Other authors hypothesize a faulty intestinal recanalization for an abnormality in the vascularization process during the sixth-seventh week of fetal life, in which the cystic spaces by several vacuoles merge into one another, but do not join the main lumen. Finally, the action of environmental stresses on the fetus, particularly trauma and hypoxia, is reported in the literature, even in different gestation periods.

The variants of intestinal duplications are formations adjacent to the gastrointestinal tract of origin with which they share the muscular lining and vascular axis, but have an autonomous mucosal lining that is not necessarily the same as the adjacent gastrointestinal segment; in fact, heterotopic islets of gastric mucosa may be present, rarely pancreatic tissue and in exceptional cases ciliate respiratory epithelium.^4^

In 80% of cases, intestinal duplication shows cystic morphology: this subtype is found predominantly in the small intestine and in particular in the terminal ileum; when placed near the ileo-cecal junction it can either cause intestinal obstruction during the neonatal period or remain asymptomatic and represent a potential point of invagination. Cystic duplications in 80% of cases do not communicate with the lumen of the adjacent intestine; they contain a clear mucoid substance secreted by the same mucosa, sometimes hemorrhagic due to the presence of gastric ectopias with mucosal ulcerations.^5^

In 20% of cases intestinal duplications have tubular morphology, they are located on the antimesenteric side—and have a common wall with the intestine and communicate with its lumen at the level of the distal extremity. Often, they are ulcerated not so much within their lumen, as in level and especially downstream of the junction with the normal bowel (peptic ulcer).

In the majority of patients, intestinal duplication is diagnosed before the age of 2, with more than half before the 6 months of life, however it is possible to find intestinal duplications in any period of life. Duplication of the alimentary canal does not present a specific symptoms, indeed it can manifest with a variety of symptoms, including distension and abdominal pain, sickness, hemorrhage, chronic respiratory disorders, abdominal painless mass, and often the onset symptoms can be confused with other gastrointestinal diseases. In general, the symptoms are related to the position, size, shape and type of mucosa present; in fact, in the presence of gastric mucosa, the probable ulceration can lead to bleeding or even perforation. Rarely they can present with acute complications such as intestinal invagination or mechanical occlusion.^6^

The differential diagnosis includes all causes of neonatal intestinal obstruction, such as intestinal invagination, omental or mesenteric cysts, pancreatic pseudocysts, esophageal hepatic cysts, and in ovarian cyst. In most cases the diagnosis is performed with prenatal ultrasound, where the sign of the “double wall” consists of a hyperechoic internal rim related to the mucosa–submucosa, an external hypoechoic layer surrounding the mucosa–submucosa, and a hypoechoic outer layer surrounding the muscle itself, as well as the presence of peristalsis. Approximately one-third of patients with enteric duplications have associated malformations, such as spinal defects, pulmonary sequestration, congenital cystic adenomatoid malformation, and cardiac defects. For this reason, the comparison with the pre-natal ultrasound of a duplication of the alimentary canal requires the pre-natal MR for precise description of the enteric duplications, and of an echocardiogram. In the post-natal period the radiography of the abdomen can reveal a mass effect; the ultrasound will allow distinction between cystic or solid mass as well as the relationship with the intestine, while the characteristic external hypoechogenicity and internal echogenicity will indicate the intestinal nature of the cyst. CT or MR can be useful in cases where ultrasound is not enlightening. Scintigraphy with ^99m^Tc *pertechnetate* can be used to identify a gastric ectopic mucosa in the cyst; this technique is particularly useful in investigations in children with bleeding, allowing a differential diagnosis with Meckel's diverticulum. It is also useful in the study of a child with asymptomatic duplication diagnosed prenatally, in which the demonstration of ectopic gastric mucosa can grant surgical treatment rather than watchful waiting. The esophagus-gastro-duodenoscopy (EGDS) can be useful to identify ulcers or stenosis and to better define the anatomy before the surgical excision.^7^

In the specific case of ileal duplications, laparoscopy has recently been proposed both for diagnosis in doubtful cases and for treatment, thus eliminating open-air exploration and decreasing the duration of postoperative admission.^8^ The treatment of small intestinal duplications is variable due to the heterogeneity of these malformations. The usual approach remains intestinal resection with primary anastomosis. Rarely, small cystic duplications can be treated with enucleation without sacrificing the native blood supply. Resections of larger bowel segment will increase complications with the risk of short bowel syndrome. In this situation, the mucosal stripping, sometimes through multiple enterotomies, will preserve the length of the intestine and will decrease the risk of ulceration and hemorrhage from the ectopic mucosa.^9^

## Learning point

- Intestinal duplication cysts are rare congenital malformations that may be asymptomatic throughout life, or present with very vague symptoms and rarely with acute symptoms.
- In the case presented, an ultrasound examination performed following a slight trauma showed ileal duplication cyst, as an incidental finding.
- The formation was removed by an ileal resection with terminolateral anastomosis and a prophylactic appendectomy avoiding a rare, but still possible, acute presentation (*e.g.* intestinal invagination).
